# Antimicrobial resistance and genomic analysis of *Vibrio parahaemolyticus* isolates from foodborne outbreaks, Huzhou, China, 2019–2023

**DOI:** 10.3389/fmicb.2024.1439522

**Published:** 2024-09-11

**Authors:** Wei Yan, Lei Ji, Fenfen Dong, Liping Chen, Rui Yuan, Peng Zhang

**Affiliations:** Huzhou Center for Disease Control and Prevention, Huzhou, Zhejiang, China

**Keywords:** *Vibrio parahaemolyticus*, foodborne disease, outbreak, PFGE, WGS

## Abstract

**Objective:**

The purpose of this study was to investigate the epidemiological and genomic characteristics of *Vibrio parahaemolyticus* (*V. parahaemolyticus*) isolates from outbreaks in Huzhou, China.

**Methods:**

This study aims to analyze the epidemiological data on *V. parahaemolyticus* outbreaks reported in Huzhou from 2019 to 2023. A total of 70 *V. parahaemolyticus* outbreak isolates were collected. The antibiotic resistance, serotypes, molecular typing, and genomic characteristics of these isolates were analyzed.

**Results:**

Most outbreaks of *V. parahaemolyticus* infection occurred in the summer, and the majority of outbreaks occurred in restaurants and rural banquets. High resistance rates were observed for ampicillin (AMP, 24.29%), followed by tetracycline (TET, 15.71%) and trimethoprim-sulfamethoxazole (SXT, 15.71%). The newly emerged serotype O10:K4 became dominant from 2021 to 2023, with most isolates belonging to ST3. The resistance gene *blaCARB* was frequently detected among these isolates. The pulsed-field gel electrophoresis (PFGE) and whole-genome single-nucleotide polymorphisms (wgSNPs) effectively differentiated the nine outbreaks.

**Conclusion:**

The newly emerged serotype O10:K4 became dominant from 2021 to 2023, with most isolates being ST3. PFGE and WGS technologies provided reliable methods for typing and identifying *V. parahaemolyticus* for outbreaks.

## 1 Introduction

*Vibrio parahaemolyticus* (*V. parahaemolyticus*) is a Gram-negative bacterium commonly found in temperate and tropical coastal areas worldwide. It is a significant foodborne pathogen, particularly prevalent in halophilic environments. Cases of *V. parahaemolyticus* infections were mostly associated with the consumption of raw or undercooked contaminated food in summer (Letchumanan et al., 2014; Martinez-Urtaza and Baker-Austin, 2020). Symptoms of acute gastroenteritis caused by *V. parahaemolyticus* include nausea, vomiting, and diarrhea, with severe cases potentially leading to death (Baker-Austin et al., 2010). *V. parahaemolyticus* was first isolated and geographically restricted to Japan in 1950, but its infections were reported in geographically diverse locations from 1969. Since 1996, the worldwide prevalence of the O3:K6 serotype, *V. parahaemolyticus*, has gradually become a major global foodborne pathogen (Hara-Kudo et al., 2012), and according to the estimates of 2020, there were over 50,000 infections each year in the United States (Martinez-Urtaza and Baker-Austin, 2020). Coastal countries such as Canada, the United States, Mexico, Spain, and South Korea have also reported cases of *V. parahaemolyticus* infections (Taylor et al., 2018; McLaughlin et al., 2005; Cabanillas-Beltrán et al., 2006; Martinez-Urtaza et al., 2016; Jeong et al., 2020).

In China, *V. parahaemolyticus* is becoming a major pathogen for bacterial foodborne outbreaks (Li W. et al., 2020), particularly in eastern coastal areas such as Shanghai and Zhejiang Province (Luo et al., 2019; Chen et al., 2022b; Chen et al., 2022a). In Shanghai, the incidence rate of *V. parahaemolyticus* infections was 183 cases per 100,000 individuals (Luo et al., 2019). The research of Zhejiang indicated that *V. parahaemolyticus* had become the main cause of foodborne outbreaks, which account for 58.4% (Chen et al., 2022b; Chen et al., 2022a). Currently, there has been an increase in foodborne outbreaks attributed to *V. parahaemolyticus* in Zhejiang province, China, with reported cases escalating from 5 in 2011 to 45 in 2021 (Chen et al., 2023).

Located on the southeast coast of China, Huzhou had a permanent population of 3.44 million at the end of 2023. The southeastern coastal region of China was considered a hotspot for *V. parahaemolyticus* prevalence (Chen et al., 2016). *V. parahaemolyticus* has emerged as the second most commonly detected pathogen in diarrhea cases in Huzhou, Zhejiang Province, followed by diarrheal *Escherichia coli* (Wu et al., 2021). There were different degrees of *V. parahaemolyticus* contamination in freshwater and seawater products in Huzhou (Yan et al., 2021). In 2010, the Foodborne Disease Outbreaks Surveillance System (FDOSS) was established. Analysis of the data revealed a consistent occurrence of foodborne outbreaks caused by *V. parahaemolyticus* since 2019 in Huzhou. However, there is a lack of reports analyzing antimicrobial resistance (AMR) profiles and genetic diversity of *V. parahaemolyticus* from foodborne outbreaks.

The aim of this study was to analyze the outbreaks caused by *V. parahaemolyticus* in Huzhou from 2019 to 2023, which were obtained from FDOSS. We conducted an investigation of the AMR profiles and molecular epidemiological characteristics of 70 *V. parahaemolyticus* isolates from foodborne outbreaks over a 5-year period. This study provides us with the genetic diversity, virulence potential, and AMR profiles of *V. parahaemolyticus* from outbreaks in Huzhou. This could be beneficial for future intervention and prevention of foodborne diseases caused by *V. parahaemolyticus.*

## 2 Materials and methods

### 2.1 Definition of *Vibrio parahaemolyticus* outbreak

The *V. parahaemolyticus* outbreak is defined as two or more cases of diarrhea (≥3 times/24 h) by consuming the same food contaminated with *V. parahaemolyticus*. The bacteria can be isolated from the food, equipment used in food preparation, stool, or vomit of multiple affected individuals.

### 2.2 Epidemiological investigation

After the outbreak, the local public health physician conducted an investigation. Suspected cases were further investigated based on a standardized questionnaire. The questionnaire included basic information (such as age and gender), clinical details (including onset time, symptoms such as vomiting, diarrhea, abdominal pain, fever, and stool character), and information on food exposure. Additionally, the sanitary investigation was carried out on-site, and food and environment samples were obtained for laboratory testing.

### 2.3 Sample collection and *Vibrio parahaemolyticus* isolation

From 2019 to 2023, there were 9 foodborne outbreaks caused by *V. parahaemolyticus* in Huzhou. A total of 218 samples were collected, comprising 151 patient samples (83 anal swabs, 62 stool samples, and 6 vomit samples), 39 food samples (21 aquatic products, 10 Chinese cold dishes, 5 meat and meat products, and 3 vegetables), and 28 environmental samples (9 bowls, 7 plates, 5 cutting boards, 5 kitchen water samples, and 2 kitchen knives). Among these samples, 70 isolates of *V. parahaemolyticus* were isolated, with 68 originating from patients and 2 from food, and no isolates were obtained from environmental samples. The isolation and identification of *V. parahaemolyticus* in each outbreak were conducted in accordance with the Chinese National Standard for Food Safety Manual (GB 4789.7–2013). For each sample, enrichment was performed using 3% sodium chloride alkaline peptone water at 37°C for 18 h. The enriched cultures were then plated on Chromogenic Vibrio agar (Chromogar, France) and incubated at 37°C for 24 h for colony screening. The single colony display purple or fuchsia coloration was selected and confirmed using the VITEK MS system (bioMérieux, France).

### 2.4 Serotyping

Serotypes of *V. parahaemolyticus* isolates were determined by slide agglutination for 11 O (lipopolysaccharide) and 65 K (capsule) antisera using commercially available antiserum (Denka Seiken, Japan). Pure cultures of bacterial strains were washed from the agar plates with 3% NaCl and 5% glycerin solution. A part of the suspension was directly mixed with K antigen antisera for slide agglutination reactions. Another part suspension was subjected to autoclave at 121°C for 1 h, followed by centrifugation at 4000 rpm for 15 min, and the supernatant was discarded and washed with normal saline three times. The final suspension was tested against antisera for O-antigens agglutinating reaction, with normal saline used as a negative control. The serotyping scheme of *V. parahaemolyticus* is based on the combination of O and K antigens.

### 2.5 Antimicrobial susceptibility testing

Antibiotic susceptibility of all *V. parahaemolyticus* isolates was tested using the broth micro-dilution minimum inhibitory concentration (MIC) method. A bacterial colony suspension was prepared in a sterile 0.9% NaCl solution and adjusted to a 0.5 McFarland standard. The suspension was added to the broth, and then the broth was inoculated onto the antimicrobial susceptibility plate and incubated for 24 h at 36°C. The 12 antibiotics selected were chloramphenicol (CHL), trimethoprim-sulfamethoxazole (SXT), meropenem (MEM), cefotaxime (CTX), ceftazidime (CAZ), tetracycline (TET), ciprofloxacin (CIP), nalidixic (NAL), azithromycin (AZM), amikacin (AMI), ampicillin (AMP), and ampicillin/sulbactam (AMS). Breakpoint interpretations were developed by reference to Clinical and Laboratory Standards Institute (CLSI) guidelines (CLSI, 2020). Negative and blank controls were set up at the same time, and *Escherichia coli* ATCC 25922 was included as a quality control strain.

### 2.6 Pulsed-field gel electrophoresis analysis

The PFGE analysis of *V. parahaemolyticus* was carried out according to the standard operation method of PulseNet International guidelines (Parsons et al., 2007). Not I (50 U/μL) was selected as the restriction enzyme that could digest the chromosomal DNA of *V. parahaemolyticus.* The gel blocks were loaded onto the 1% SeaKem Gold agarose gel (Lonza, Swiss) and subjected to PFGE testing using the CHEF Mapper pulsed-field electrophoresis system (Bio-Rad, United States). The electrophoresis conditions were set as follows: the electrophoresis time was 18 h, the initial switch time of 10 s, and the final switch time of 35 s. The gel image was observed after GelRed staining and subsequent decolorization with pure water. BioNumerics software 7.6 (Applied Maths, Belgium) was used to analyze the images. Clustering was conducted using the unweighted pair group method (UPGMA) and the dice correlation coefficient with a position tolerance of 1.5%. Bands with a similarity greater than 85% were grouped into the same gene cluster (Tenover et al., 1995).

### 2.7 Whole-genome sequencing and genomic analysis

The DNA of *V. parahaemolyticus* isolates was extracted using the QIAamp DNA Mini Kit (Qiagen, Germany) according to the manufacturer’s instructions. The concentration of *V. parahaemolyticus* DNA was tested using the Qubit 4 (Thermo, United States), and the qualified DNA was stored at −80°C for further use. WGS libraries were constructed using the Metagenomic DNA Library Kit (Matridx, China) and subsequently sequenced on the NextSeq 550 High Output Reagent Cartridge v2 300 cycles (Illumina, United States).

Raw data were assessed for quality, trimmed, and subsequently assembled into a draft genome sequence using the SPAdes software. For multilocus sequence typing (MLST), we submitted the sequence of isolates to the PubMLST for housekeeping genes (*dnaE, gyrB, recA, dtdS, pntA, pyrC,* and *tnaA*) allele alignment.^1^ The antibiotic resistance genes of the isolates were predicted using ResFinder.^2^ Virulence genes were detected via the virulent factors of pathogenic bacteria (VFDB) database.^3^ Considering GCA_000196095.1 as a reference genome, we used Snippy to obtain the wgSNPs of the isolates. FastTree was used for sequence alignment and homology analysis, and Gubbins was used for reassembly. Finally, the phylogenetic tree and the heatmap of genes were visualized using the ChiPlot tool.^4^ The genome sequences had been submitted to GenBank. The BioSamples of NCBI are from SAMN41518030 to SAMN41518099.

## 3 Results

### 3.1 Epidemiological characteristics

From 2019 to 2023, the Huzhou Center for Disease Control and Prevention (CDC) reported 9 outbreaks caused by *V. parahaemolyticus*, resulting in 212 illnesses and 5 hospitalizations (Table 1). The median number of patients in all outbreaks was 18 (range: 5–59). The incidence of *V. parahaemolyticus* outbreaks exhibits a distinct seasonality, with the onset time being concentrated from May to September. Specifically, 66.67% of all outbreaks (6/9) and 72.17% of total cases (153/212) were reported in July and August. *V. parahaemolyticus* outbreaks were most commonly reported in restaurants and rural banquets, accounting for 44.44%, respectively. The largest recorded *V. parahaemolyticus* outbreak occurred in a restaurant, resulting in 59 cases.

**Table 1:** Epidemiological characteristics of nine *Vibrio parahaemolyticus* outbreaks.

Outbreak time	Cases(n)	Hospitalizations(n)	Numbers of isolates(n)	Setting
2019.7	41	2	6	Restaurant
2020.8	5	2	2	Household
2021.6	28	0	17	Rural banquet
2021.8	59	1	6	Restaurant
2022.7	26	0	16	Rural banquet
2022.9	18	0	8	Rural banquet
2023.5	13	0	3	Rural banquet
2023.7	10	0	4	Restaurant
2023.8	12	0	8	Restaurant

The main symptoms of the cases were diarrhea (99.06%, 210/212), abdominal pain (88.68%, 188/212), vomiting (53.30%, 113/212), fever (41.51%, 88/212), and headache (34.91%, 74/212). In all reported outbreaks, the maximum incubation period was 36 h and the minimum was only 2 h.

### 3.2 Serotyping and MLST

Only 4 serotypes were identified among the 70 *V. parahaemolyticus* isolates, with O10:K4 being the dominant type, accounting for 61.43% (43/70) of the isolates, followed by serotypes O4:KUT (20.00%, 14/70) and O3:K6 (15.71%, 11/70). A total of 54 *V. parahaemolyticus* isolates were fully typed, resulting in a complete typing rate of 77.14%. Additionally, 16 isolates had untyped K antigens that were O4:KUT and O5:KUT (22.86%, 16/70). Both O5: KUT strains were isolated from food samples from the same outbreak. MLST revealed 2 sequence types (STs) among 68 strains, specifically ST3 and ST2516.

### 3.3 Antimicrobial susceptibility

All 70 isolates of *V. parahaemolyticus* were tested for AST using 7 antibiotic groups (Table 2), including β-lactam, fluoroquinolones, macrolides, aminoglycosides, tetracyclines, amphenicol, and sulfonamides. A total of 24 *V. parahaemolyticus* isolates (34.29%) demonstrated susceptibility to all 12 tested antimicrobials. Notably, all isolates exhibited sensitivity to MEM, AZM, and AMI. The highest resistance rate was recorded for AMP (24.29%), followed by TET (15.71%) and SXT (12.86%). In contrast, MEM, AZM, AMI, and CHL exhibited a 0.00% resistance rate. Only one *V. parahaemolyticus* isolate displayed resistance to three or more antimicrobials, with a multi-drug resistance (MDR) spectrum of AMP-AMS-NAL-TET.

**Table 2:** Antimicrobial susceptibilities of 70 *V. parahaemolyticus* isolates.

Class	Antimicrobial	Sensitive(n,%)	Intermediate(n,%)	Resistant(n,%)
β-lactam	AMP	43(61.43)	10(14.28)	17(24.29)
	AMS	60(85.71)	8(11.43)	2(2.86)
	CAZ	68(97.14)	0(0.00)	2(2.86)
	CTX	68(97.14)	0(0.00)	2(2.86)
	MEM	70(100.00)	0(0.00)	0(0.00)
quinolones	NAL	66(94.29)	0(0.00)	4(5.71)
	CIP	67(95.71)	1(1.43)	2(2.86)
macrolides	AZM	70(100.00)	0(0.00)	0(0.00)
aminoglycosides	AMI	70(100.00)	0(0.00)	0(0.00)
tetracyclines	TET	59(84.29)	0(0.00)	11(15.71)
amphenicol	CHL	68(97.14)	2(2.86)	0(0.00)
sulfonamides	SXT	61(23.26)	0(0.00)	9(12.86)

### 3.4 PFGE analysis

**Figure 1:**
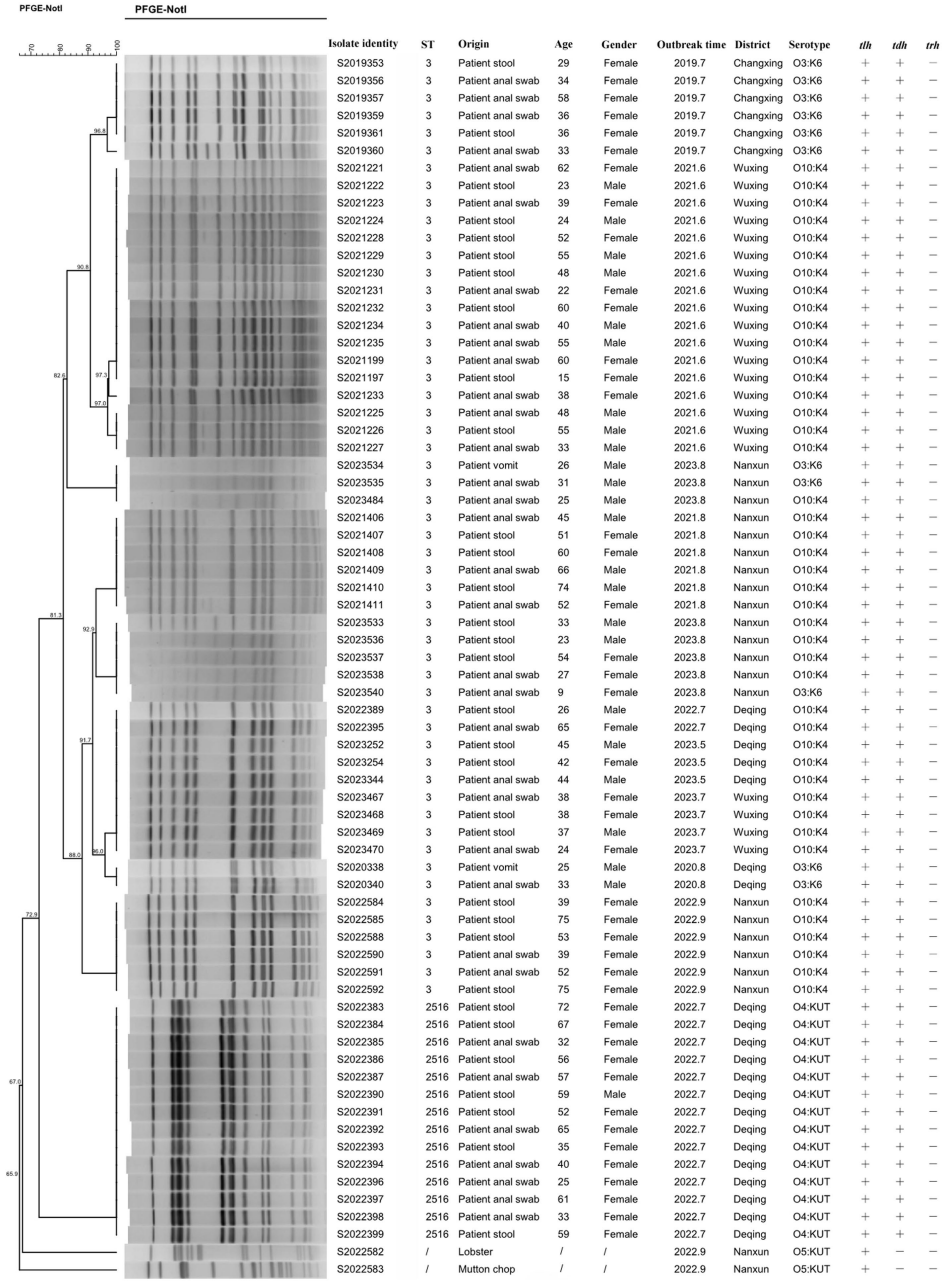
PFGE pattern of 70 *Vibrio parahaemolyticus* isolates from outbreaks.

PFGE analysis was performed using the restriction enzyme Not I to study the genetic relatedness among *V. parahaemolyticus* isolates. This analysis revealed 14 distinguishable patterns among the 70 isolates, with similarity ranging from 65.9 to 100.0% (Figure 1). These isolates showed a tendency to cluster on the basis of their STs and serotype profiles, although some O3:K6 isolates mixed with O10:K4 and showed high similarity with O10:K4. Given that all isolates were obtained from outbreaks, the PFGE patterns were relatively concentrated. Using an 85% similarity cutoff value, the 68 isolates were divided into 4 genetic clusters, while the remaining 2 isolates exhibited dispersed patterns. Notably, each outbreak had two or more strains with the same pattern, showing a similarity of 100.0%.

### 3.5 Detection of antibiotic-resistant genes and virulence factors

After comparison and analysis with the ResFinder database, it was found that 70 isolates of *V. parahaemolyticus* contained *blaCARB*, with *blaCARB-22* being the most prevalent (77.14%, 54/70), followed by *blaCARB-20* (20.00%, 14/70), *blaCARB-26* (1.43%, 1/70), and *blaCARB-46* (1.43%, 1/70). The *blaCARB* was one of the β-lactam genes that could cause antibiotic inactivation to acquire drug resistance, with the *blaCARB* point mutation, associated with resistance to amoxicillin, AMP, and piperacillin.

**Figure 2:**
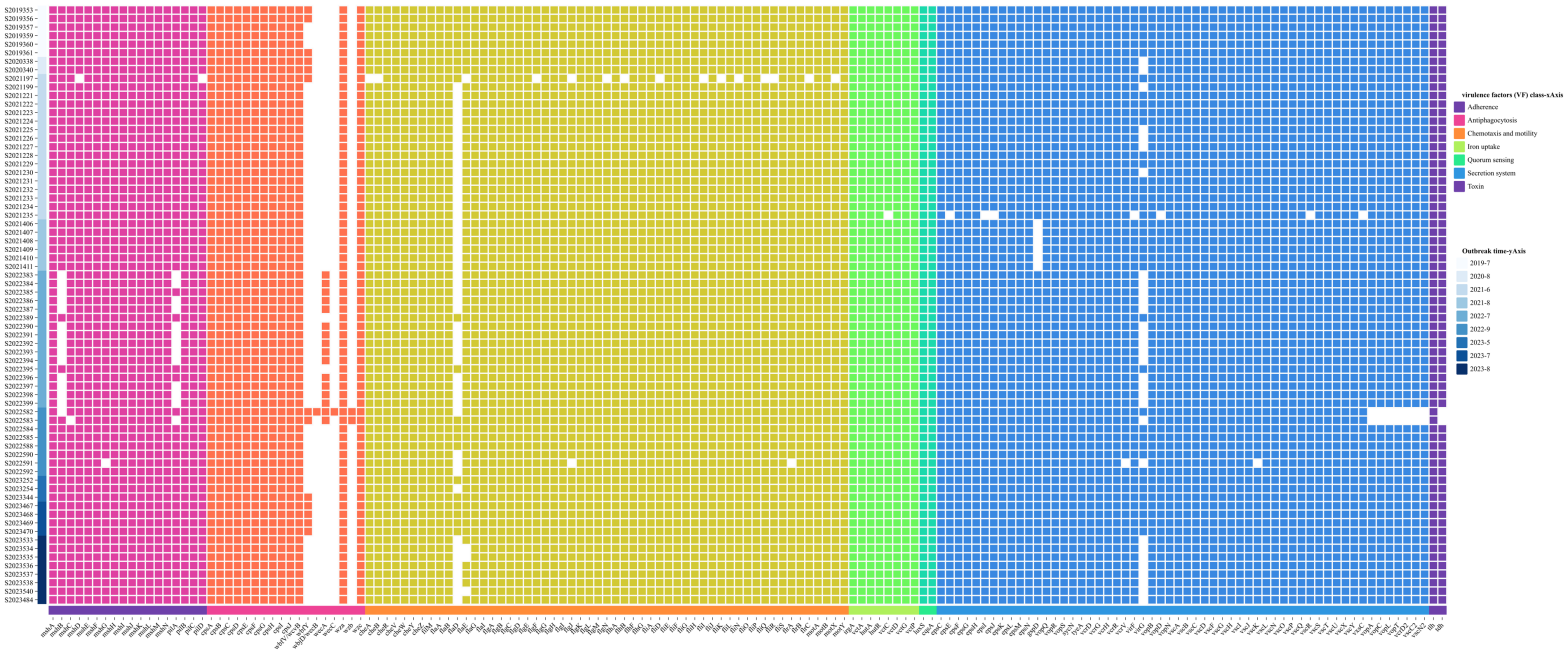
Annotation heatmap of virulence factors among 70 *V. parahaemolyticus* isolates from outbreaks.

In addition, virulence analysis using the VFDB database identified 159 virulence genes from 70 isolates. These genes were categorized into seven categories based on the roles in pathogenesis by virulence factor (VF) class, including adherence, antiphagocytosis, chemotaxis and motility, iron uptake, quorum sensing, secretion system, and toxin. The distribution of VFs is shown in Figure 2.

### 3.6 Phylogenetic analysis

**Figure 3:**
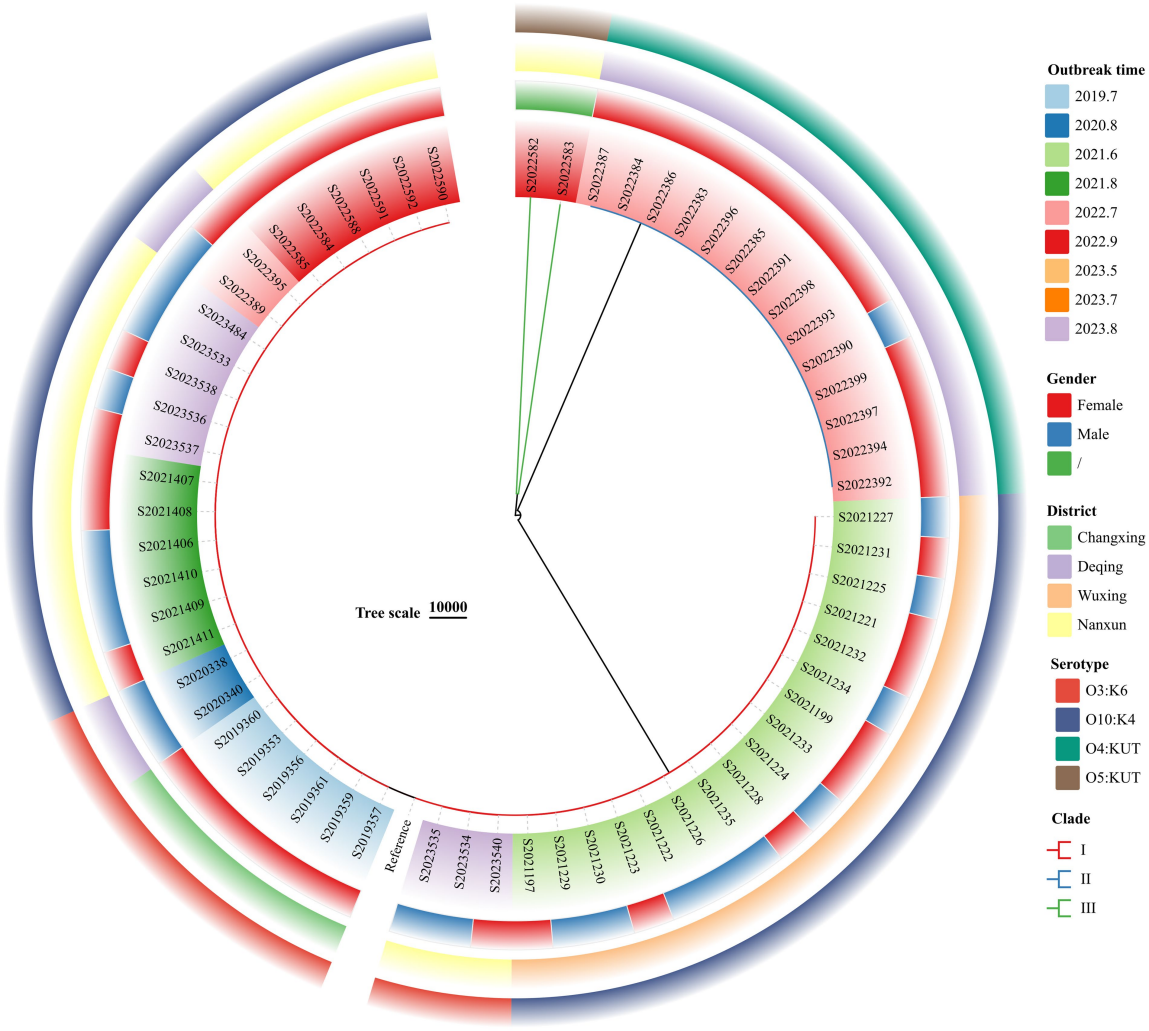
Phylogenetic analysis of all 70 *V. parahaemolyticus* isolates from outbreaks, 2019–2023.

**Figure 4:**
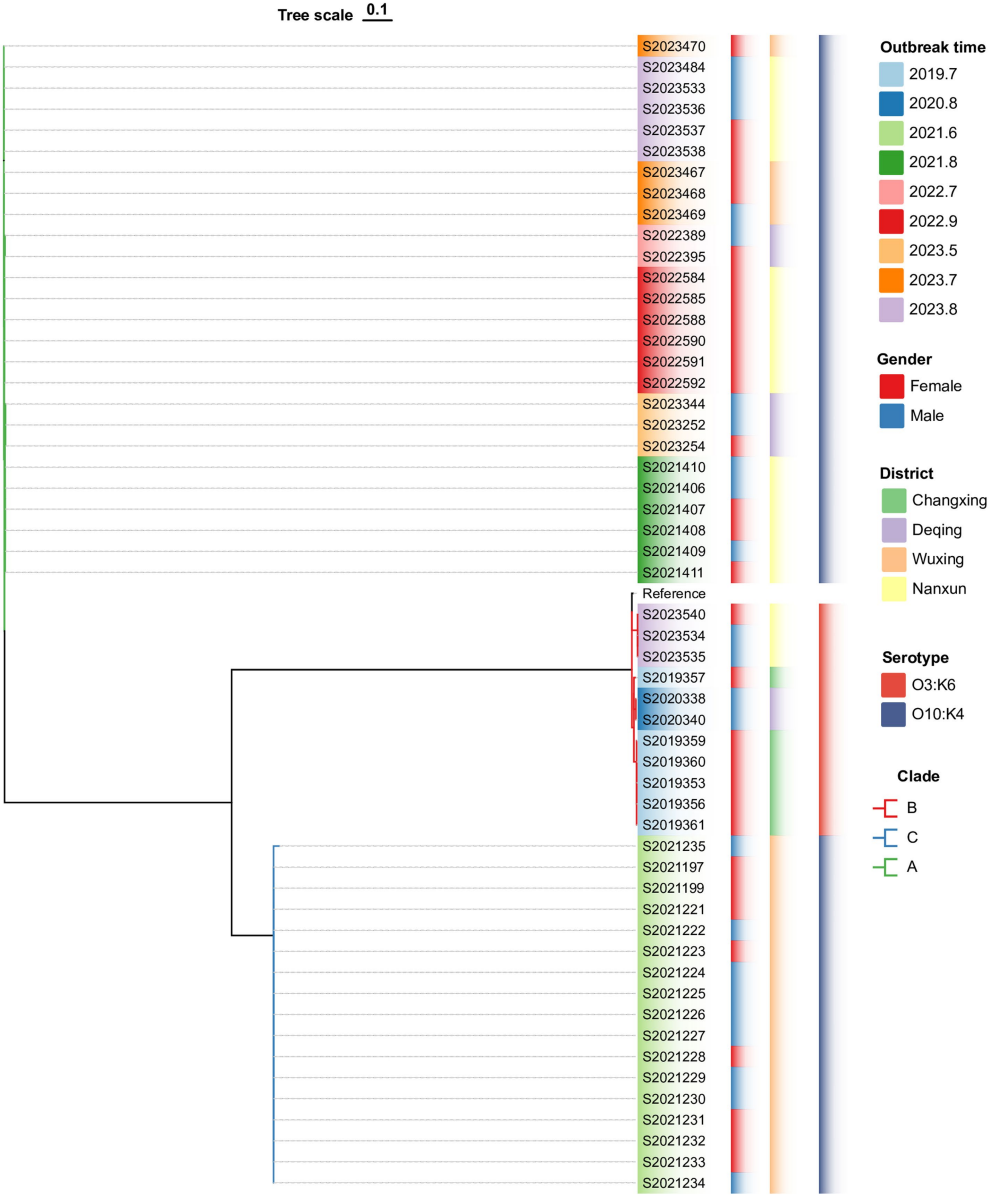
Phylogenetic analysis of 54 *V. parahaemolyticus* isolates from clade I.

The wgSNP phylogenetic analysis of 70 *V. parahaemolyticus* isolates identified three major clades, designated I, II, and III (Figure 3). These clades showed a significant correlation with the serotypes. The nine outbreaks could be clearly distinguished using wgSNP phylogenetic analysis, although there were no distinct characteristics in terms of sex and regional distribution among the cases. Clade I, the largest clade, consisted of 54 isolates, while clade II (O4:KUT) had 14 isolates, and clade III (O5:KUT) had 2 isolates originating from food and forming a single lineage. The serotyping of O3:K6 and O10:K4 concentrated in clade I was indistinguishable, leading to a reanalysis of the sequences within clade I using wgSNP (Figure 4). The reanalysis revealed that the 54 isolates in clade I could be further divided into 3 clusters (A, B, and C), with 17 isolates from an outbreak in June 2021 forming a distinct lineage known as lineage C.

## 4 Discussion

*V. parahaemolyticus* has become one of the major causes of global diarrheal diseases, and it is of great public health significance for China to perform an infectious disease surveillance project that includes *V. parahaemolyticus* (Martinez-Urtaza and Baker-Austin, 2020). In recent years, outbreaks of foodborne diseases caused by *V. parahaemolyticus* have been common in coastal provinces such as Guangdong (Li et al., 2022) and Fujian (Yuxian et al., 2023). Since 2015, *V. parahaemolyticus* has become the most important pathogen of bacterial foodborne outbreaks in our province (Chen et al., 2022b; Chen et al., 2022a). Similarly, the situation in our city is also not optimistic, with a total of nine foodborne outbreaks caused by *V. parahaemolyticus* reported in Huzhou over the past 5 years; more than one outbreak occurred every year. In particular, three *V. parahaemolyticus* outbreaks were recorded in 2023 alone. *V. parahaemolyticus* has increasingly become a significant risk factor to food safety in Huzhou.

From 2019 to 2023, we collected 70 isolates of *V. parahaemolyticus* from outbreaks in Huzhou, analyzing their antimicrobial susceptibility and genomic characteristics. These outbreaks caused by *V. parahaemolyticus* had obvious seasonal characteristics, with all nine outbreaks occurring between May and September when the temperature increased, which was consistent with many studies (Chen et al., 2023; Wang et al., 2015; Park et al., 2018). Similarly, most reports of *V. parahaemolyticus* infection cases were predominantly observed in the summer (Huang et al., 2023; Zhang et al., 2022). This is because of the mesothermal nature of *V. parahaemolyticus*, which results in higher concentrations of *V. parahaemolyticus* in aquatic products or seawater during summer compared to the other seasons (Cook et al., 2002; Li Y. et al., 2020). Gastroenteritis associated with *V. parahaemolyticus* infection is typically self-limiting and resolves within 72 h (Baker-Austin et al., 2010), as a result, only 5 of the 212 patients were hospitalized. Aquatic products, especially seafood, were the foods responsible for most *V. parahaemolyticus* outbreaks. *V. parahaemolyticus* has also been isolated from freshwater aquatic products, even some ready-to-eat foods (cooked meat and cold dishes; Li et al., 2019; Xie et al., 2016). Given that *V. parahaemolyticus* outbreaks occur more frequently in restaurants and rural banquets, it is important to implement enhanced supervision and specialized training programs for chefs on seafood preparation in order to effectively mitigate the risk of *V. parahaemolyticus*.

Before 2021, the predominant serotype of *V. parahaemolyticus* outbreaks in Zhejiang Province was O3:K6 (Chen et al., 2023; Yan et al., 2020); however, our previous research (Zhang et al., 2022) has shown that the main serotype of sporadic clinical patients had shifted to O10:K4 from 2021 in Huzhou. In this previous research, we found that O10:K4 was detected in Huzhou sporadically in 2020 (13.58%, 11/81); however, O10:K4 suddenly increased to 77.50% (62/80) in 2021, becoming the dominant serotype instead of O3:K6. This study about *V. parahaemolyticus* outbreaks also confirms this viewpoint. The outbreaks in 2019 and 2020 were caused by the O3:K6 serotype, and after 2020, the outbreaks began to be caused by the O10:K4 serotype. The emergence of new serum variants poses a challenge to the prevention and control of outbreaks and epidemics of *V. parahaemolyticus* in the future. This requires us to re-understand and analyze the characteristics of new serotypes. The mechanism behind the continuous emergence of new serotypes and the relationship between the different serotypes require further study. The isolates in all 9 outbreaks were associated with ST3, and ST3 dominated in 8 of them, consistent with other reports (Han et al., 2016; He et al., 2021). Another outbreak was mainly ST2516, which was suspected to be the ST345 clone group, with two differing only in the *recA* gene. The *recA* variation is closely related to the genetic evolution of *V. parahaemolyticus* (Han et al., 2014), and disease prevention and control still requires vigilance against infection caused by rare ST (such as ST2516).

Although infections associated with *V. parahaemolyticus* are often self-limiting, antibiotic therapy is sometimes used if infections do not resolve or progress to systemic infections. The rapid increase in antimicrobial resistance (Baker-Austin et al., 2010) of *V. parahaemolyticus* has become a serious public health concern. With the irrational use of antibiotics in clinical and other fields, the problem of resistance is becoming more and more serious (Arias and Murray, 2009). Antibiotic treatment recommendations for *V. parahaemolyticus* infections include TET, fluoroquinolones (CIP), third-generation cephalosporins (CTX), aminoglycosides (AMI), and folate pathway inhibitors (SXT; Shaw et al., 2014). Fortunately, in our study, with the exception of TET (15.71%) and SXT (12.86%), which have relatively high resistance rates, the remaining recommended antibiotics all have resistance rates below 3.00%. The AST results revealed a high level of resistance (24.29%, 17/70) of the outbreak isolates of *V. parahaemolyticus* to AMP, which aligns with the high resistance rate of AMP in *V. parahaemolyticus* reported in different regions worldwide. A study in Vietnam reported that 81.4% of seafood isolates demonstrated resistance to AMP (Vu et al., 2022). Similarly, Obaidat et al. (2017) reported an AMP resistance rate of 43.0% among imported fish isolates from Jordan. The high rates of AMP resistance have been observed in clinical isolates from Nantong and Huzhou in China, reaching 64.2 and 86.8%, respectively (Huang et al., 2023; Zhang et al., 2022). These indicate that AMP may be ineffective for the treatment of *V. parahaemolyticus* infections. In this study, MDR was rare, and only one multi-drug-resistant isolate was found in 70 isolates. This may be related to the small sample size we studied.

PFGE is the gold standard for molecular traceability because of its high resolution, good repeatability, and easy standardization (Carstens et al., 2019). In this study, the PFGE fingerprint patterns revealed a high degree of homogeneity among strains from the same outbreak within the same genetic cluster; thus, we can judge whether the strains have homology by the similarities between PFGE patterns. The PFGE can also determine the genetic relatedness between *V. parahaemolyticus* isolates with serotypes O3:K6 and O10:K4. It could be found that the O3:K6 serotype was mixed with the O10:K4 serotype, which means that the two serotypes were genetically similar. Zhao et al. (2019) reported that divergent serotypes could be related to genes. The changes in serotype by mutation or horizontal gene transfer may be a pattern for *V. parahaemolyticus* to adapt to circumstance variation and human immune systems (Mahmud et al., 2007). Similarly, PFGE could distinguish among different ST types very well, with ST3 and ST2516 concentrated in different regions of Figure 1. There may be two or more PFGE patterns of isolates in the same outbreak, suggesting the possibility of simultaneous infection of multiple cloned isolates in the same outbreak (2022.9 and 2023.8).

All isolates in this study carried the β-lactam resistance gene *blaCARB*, but the resistance rate of AMP was only 24.29%. It demonstrated that some genes are usually not sufficient to cause changes in the resistance phenotype unless the strain has mutations that can increase their expression (Salipante and Hall, 2003). Thermostable direct hemolysin (TDH) and TDH-related hemolysin (TRH) are considered to be the most important VFs of *V. parahaemolyticus*, which can form 2 nm pores on the cell membrane, allowing water and ions to enter freely, thus making the red blood cells have osmotic dissolution and hemolytic activity, and they share 70% genetic homology (Raghunath, 2015). It is generally believed that clinical isolates mostly contain the virulence gene *tdh*, while food isolates mainly carry the virulence gene *trh* (Raghunath, 2015). Most infections are associated with isolates that possess *tdh* (Ottaviani et al., 2012). Type III secretion systems (T3SSs) are important VFs from the secretion system. T3SS factors contain more than 40 factors, such as *Vop Q* and *Vop L*. Their function is to mediate the translocation of bacterial effector proteins directly into eukaryotic cells (Portaliou et al., 2016) and ensure *V. parahaemolyticus* survival in the environment (Domman et al., 2017). *V. parahaemolyticus* could use adhesion factors to bind to fibronectin on the host cell and use T3SSs to transport different effectors and toxins into the cell, causing serious diseases.

WGS can rapidly generate a large amount of precise data, which is used for species identification, determining virulence and resistance characteristics, and conducting phylogenetic analyses (Ribot et al., 2019). With the decrease in sequencing cost, WGS has been gradually applied to the surveillance of foodborne pathogens. SNP analysis based on WGS, as a new molecular typing method, is replacing PFGE in outbreak tracing (Taylor et al., 2015). In this study, wgSNP phylogenetic analysis revealed that the *V. parahaemolyticus* from outbreaks in Huzhou encompasses a wide range of genotypes that can be divided into three main clades; however, it is able to clearly distinguish the nine outbreaks. Isolates with the same outbreak are more closely related and carry more consistent VFs. Using wgSNPs analysis, we can directly compare the differences of SNP mutation sites between isolates to determine whether it is the same outbreak.

The major limitations of this study were that we analyzed only outbreak strains of *V. parahaemolyticus*. Further studies are required with a greater detection range of sporadic strains to evaluate the effects of *V. parahaemolyticus*-related diseases and provide a scientific basis for the prevention and control of *V. parahaemolyticus* infection in Huzhou.

## 5 Conclusion

This study is one of the most extensive genomic analyses of *V. parahaemolyticus* from outbreaks in Huzhou, offering valuable insights into the prevalence of epidemiological characteristics, AST, STs, serotypes, PFGE analysis, virulence factors, and phylogenetic relationships from 2019 to 2023. *V. parahaemolyticus* outbreaks had obvious seasonal characteristics, and the majority of outbreaks occurred in restaurants and rural banquets. The high resistance rates to AMP (24.29%), with the following being TET (15.71%) and SXT (15.71%). The newly emerged serotype O10:K4 became dominant from 2021 to 2023, and most of the isolates were ST3. WgSNP analysis based on WGS technology provided reliable methods for the identification of the pathogens for outbreaks, caused by *V. parahaemolyticus*.

## Data Availability

The datasets presented in this study can be found in online repositories. The names of the repository/repositories and accession number(s) can be found at: https://www.ncbi.nlm.nih.gov/genbank/, SAMN41518030.

## Footnotes

^1^ https://pubmlst.org/organisms/vibrio-parahaemolyticus

^2^ http://genepi.food.dtu.dk/resfinder

^3^ http://www.mgc.ac.cn/VFs/search_VFs.htm

^4^ https://www.chiplot.online/

## References

[ref1] Arias C. A. Murray B. E. (2009). Antibiotic-resistant bugs in the 21st century--a clinical super-challenge. N. Engl. J. Med. 360, 439–443. doi: 10.1056/NEJMp0804651, PMID: 19179312

[ref2] Baker-Austin C. Stockley L. Rangdale R. Martinez-Urtaza J. (2010). Environmental occurrence and clinical impact of Vibrio vulnificus and *Vibrio parahaemolyticus*: a European perspective. Environ. Microbiol. Rep. 2, 7–18. doi: 10.1111/j.1758-2229.2009.00096.x 23765993

[ref3] Cabanillas-Beltrán H. Llausás-Magaña E. Romero R. Espinoza A. García-Gasca A. Nishibuchi M.. (2006). Outbreak of gastroenteritis caused by the pandemic *Vibrio parahaemolyticus* O3:K6 in Mexico. FEMS Microbiol. Lett. 265, 76–80. doi: 10.1111/j.1574-6968.2006.00475.x 17107421

[ref4] Carstens C. K. Salazar J. K. Darkoh C. (2019). Multistate outbreaks of foodborne illness in the United States associated with fresh produce from 2010 to 2017. Front. Microbiol. 10:2667. doi: 10.3389/fmicb.2019.02667, PMID: 31824454 PMC6883221

[ref5] Chen Y. Chen X. Yu F. Wu M. Wang R. Zheng S.. (2016). Serology, virulence, antimicrobial susceptibility and molecular characteristics of clinical *Vibrio parahaemolyticus* strains circulating in southeastern China from 2009 to 2013. Clin. Microbiol. Infect. 22, 258.e9–258.e16. doi: 10.1016/j.cmi.2015.11.003 26597222

[ref6] Chen L. Sun L. Zhang R. Liao N. Qi X. Chen J. (2022a). Surveillance for foodborne disease outbreaks in Zhejiang Province, China, 2015-2020. BMC Public Health 22:135. doi: 10.1186/s12889-022-12568-4 35045858 PMC8769373

[ref7] Chen L. Wang J. Chen J. Zhang R. Zhang H. Qi X.. (2023). Epidemiological characteristics of *Vibrio parahaemolyticus* outbreaks, Zhejiang, China, 2010-2022. Front. Microbiol. 14:1171350. doi: 10.3389/fmicb.2023.1171350 37448578 PMC10336542

[ref8] Chen L. Wang J. Zhang R. Zhang H. Qi X. He Y.. (2022b). An 11-year analysis of bacterial foodborne disease outbreaks in Zhejiang Province, China. Food Secur. 11:2382. doi: 10.3390/foods11162382, PMID: 36010382 PMC9407109

[ref560] CLSI (2020). Performance Standards for Antimicrobial Susceptibility Testing. 30th Edition (Wayne, PA, USA: Clinical and Laboratory Standard Institute). Available at: https://clsi.org/standards/products/microbiology/documents/m100/. CLSI Supplement M100.

[ref9] Cook D. W. Bowers J. C. DePaola A. (2002). Density of total and pathogenic (tdh+) *Vibrio parahaemolyticus* in Atlantic and gulf coast molluscan shellfish at harvest. J. Food Prot. 65, 1873–1880. doi: 10.4315/0362-028x-65.12.1873, PMID: 12495004

[ref10] Domman D. Quilici M. L. Dorman M. J. Njamkepo E. Mutreja A. Mather A. E.. (2017). Integrated view of *Vibrio cholerae* in the Americas. Science 358, 789–793. doi: 10.1126/science.aao2136, PMID: 29123068

[ref11] Han D. Tang H. Lu J. Wang G. Zhou L. Min L.. (2014). Population structure of clinical *Vibrio parahaemolyticus* from 17 coastal countries, determined through multilocus sequence analysis. PLoS One 9:e107371. doi: 10.1371/journal.pone.0107371 25225911 PMC4165897

[ref12] Han C. Tang H. Ren C. Zhu X. Han D. (2016). Sero-prevalence and genetic diversity of Pandemic *V. parahaemolyticus* strains occurring at a global scale. Front. Microbiol. 7:567. doi: 10.3389/fmicb.2016.00567, PMID: 27148244 PMC4840284

[ref13] Hara-Kudo Y. Saito S. Ohtsuka K. Yamasaki S. Yahiro S. Nishio T.. (2012). Characteristics of a sharp decrease in *Vibrio parahaemolyticus* infections and seafood contamination in Japan. Int. J. Food Microbiol. 157, 95–101. doi: 10.1016/j.ijfoodmicro.2012.04.019 22583518

[ref14] He M. Lei T. Jiang F. Zhang J. Zeng H. Wang J.. (2021). Genetic diversity and population structure of *Vibrio parahaemolyticus* isolated from clinical and food sources. Front. Microbiol. 12:708795. doi: 10.3389/fmicb.2021.708795, PMID: 34385993 PMC8353399

[ref15] Huang A. Wang Y. Xu H. Jin X. Yan B. Zhang W. (2023). Antibiotic resistance and epidemiology of *Vibrio parahaemolyticus* from clinical samples in Nantong, China, 2018-2021. Infect Drug Resist. 16, 7413–7425. doi: 10.2147/IDR.S432197 38077299 PMC10705721

[ref16] Jeong H. W. Kim J. A. Jeon S. J. Choi S. S. Kim M. K. Yi H. J.. (2020). Prevalence, antibiotic-resistance, and virulence characteristics of *Vibrio parahaemolyticus* in restaurant fish tanks in Seoul. South Korea. Foodborne Pathog Dis. 17, 209–214. doi: 10.1089/fpd.2019.2691, PMID: 31692375

[ref17] Letchumanan V. Chan K. G. Lee L. H. (2014). *Vibrio parahaemolyticus*: a review on the pathogenesis, prevalence, and advance molecular identification techniques. Front. Microbiol. 5:705. doi: 10.3389/fmicb.2014.00705 25566219 PMC4263241

[ref18] Li B. S. Li Z. C. Liang J. H. (2022). Epidemiological and etiological characteristics of *Vibrio parahaemolyticus* strains causing foodborne disease outbreaks in Guangdong Province from 2017 to 2020. Chin J Prev Med 56, 443–447. doi: 10.3760/cma.j.cn112150-20210423-00404, PMID: 35488540

[ref19] Li Y. Pei X. Yan J. Liu D. Zhang H. Yu B.. (2019). Prevalence of foodborne pathogens isolated from retail freshwater fish and shellfish in China. Food Control 99, 131–136. doi: 10.1016/j.foodcont.2018.12.024

[ref20] Li W. Pires S. M. Liu Z. Ma X. Liang J. Jiang Y.. (2020). Surveillance of foodborne disease outbreaks in China, 2003-2017. Food Control 118:107359. doi: 10.1016/j.foodcont.2020.107359 PMC7125948 32288325

[ref21] Li Y. Xie T. Pang R. Wu Q. Zhang J. Lei T.. (2020). Food-borne *Vibrio parahaemolyticus* in China: prevalence, antibiotic susceptibility, and genetic characterization. Front. Microbiol. 11:1670. doi: 10.3389/fmicb.2020.01670 32765472 PMC7378779

[ref22] Luo L. Gu Y. Wang X. Zhang Y. Zhan L. Liu J.. (2019). Epidemiological and clinical differences between sexes and pathogens in a three-year surveillance of acute infectious gastroenteritis in Shanghai. Sci. Rep. 9:9993. doi: 10.1038/s41598-019-46480-6 31292502 PMC6620335

[ref23] Mahmud Z. H. Neogi S. B. Kassu A. Wada T. Islam M. S. Nair G. B.. (2007). Seaweeds as a reservoir for diverse *Vibrio parahaemolyticus* populations in Japan. Int. J. Food Microbiol. 118, 92–96. doi: 10.1016/j.ijfoodmicro.2007.05.009, PMID: 17629976

[ref24] Martinez-Urtaza J. Baker-Austin C. (2020). Vibrio parahaemolyticus. Trends Microbiol. 28, 867–868. doi: 10.1016/j.tim.2020.02.008 32931744

[ref25] Martinez-Urtaza J. Powell A. Jansa J. Rey J. L. Montero O. P. Campello M. G.. (2016). Epidemiological investigation of a foodborne outbreak in Spain associated with U.S. west coast genotypes of *Vibrio parahaemolyticus*. Springerplus 5:87. doi: 10.1186/s40064-016-1728-1, PMID: 26848427 PMC4729754

[ref26] McLaughlin J. B. DePaola A. Bopp C. A. Martinek K. A. Napolilli N. P. Allison C. G.. (2005). Outbreak of *Vibrio parahaemolyticus* gastroenteritis associated with Alaskan oysters. N. Engl. J. Med. 353, 1463–1470. doi: 10.1056/NEJMoa051594 16207848

[ref27] Obaidat M. M. Salman A. E. B. Roess A. A. (2017). Virulence and antibiotic resistance of *Vibrio parahaemolyticus* isolates from seafood from three developing countries and of worldwide environmental, seafood, and clinical isolates from 2000 to 2017. J. Food Prot. 80, 2060–2067. doi: 10.4315/0362-028X.JFP-17-156 29154715

[ref28] Ottaviani D. Leoni F. Serra R. Serracca L. Decastelli L. Rocchegiani E.. (2012). Nontoxigenic *Vibrio parahaemolyticus* strains causing acute gastroenteritis. J. Clin. Microbiol. 50, 4141–4143. doi: 10.1128/JCM.01993-12 23052317 PMC3502970

[ref29] Park K. Mok J. S. Ryu A. R. Kwon J. Y. Ham I. T. Shim K. B. (2018). Occurrence and virulence of *Vibrio parahaemolyticus* isolated from seawater and bivalve shellfish of the Gyeongnam coast, Korea, in 2004–2016. Mar. Pollut. Bull. 137, 382–387. doi: 10.1016/j.marpolbul.2018.10.033 30503447

[ref30] Parsons M. B. Cooper K. L. Kubota K. A. Puhr N. Simington S. Calimlim P. S.. (2007). PulseNet USA standardized pulsed-field gel electrophoresis protocol for subtyping of *Vibrio parahaemolyticus*. Foodborne Pathog. Dis. 4, 285–292. doi: 10.1089/fpd.2007.0089, PMID: 17883312

[ref31] Portaliou A. G. Tsolis K. C. Loos M. S. Zorzini V. Economou A. (2016). Type III secretion: building and operating a remarkable Nanomachine. Trends Biochem. Sci. 41, 175–189. doi: 10.1016/j.tibs.2015.09.005, PMID: 26520801

[ref32] Raghunath P. (2015). Roles of thermostable direct hemolysin (TDH) and TDH-related hemolysin (TRH) in *Vibrio parahaemolyticus*. Front. Microbiol. 5:805. doi: 10.3389/fmicb.2014.00805, PMID: 25657643 PMC4302984

[ref33] Ribot E. M. Freeman M. Hise K. B. Gerner-Smidt P. (2019). PulseNet: entering the age of next-generation sequencing. Foodborne Pathog. Dis. 16, 451–456. doi: 10.1089/fpd.2019.2634, PMID: 31241352 PMC6653803

[ref34] Salipante S. J. Hall B. G. (2003). Determining the limits of the evolutionary potential of an antibiotic resistance gene. Mol. Biol. Evol. 20, 653–659. doi: 10.1093/molbev/msg074 12679553

[ref35] Shaw K. S. Rosenberg Goldstein R. E. He X. Jacobs J. M. Crump B. C. Sapkota A. R. (2014). Antimicrobial susceptibility of Vibrio vulnificus and *Vibrio parahaemolyticus* recovered from recreational and commercial areas of Chesapeake Bay and Maryland coastal bays. PLoS One 9:e89616. doi: 10.1371/journal.pone.0089616, PMID: 24586914 PMC3934932

[ref36] Taylor M. Cheng J. Sharma D. Bitzikos O. Gustafson R. Fyfe M.. (2018). Outbreak of *Vibrio parahaemolyticus* associated with consumption of raw oysters in Canada, 2015. Foodborne Pathog. Dis. 15, 554–559. doi: 10.1089/fpd.2017.2415, PMID: 29958009

[ref37] Taylor A. J. Lappi V. Wolfgang W. J. Lapierre P. Palumbo M. J. Medus C.. (2015). Characterization of foodborne outbreaks of *Salmonella Enterica* serovar enteritidis with whole-genome sequencing single nucleotide polymorphismbased analysis for surveillance and outbreak detection. J. Clin. Microbiol. 53, 3334–3340. doi: 10.1128/JCM.01280-15, PMID: 26269623 PMC4572550

[ref38] Tenover F. C. Arbeit R. D. Goering R. V. Mickelsen P. A. Murray B. E. Persing D. H.. (1995). Interpreting chromosomal DNA restriction patterns produced by pulsed-field gel electrophoresis: criteria for bacterial strain typing. J. Clin. Microbiol. 33, 2233–2239. doi: 10.1128/jcm.33.9.2233-2239.1995, PMID: 7494007 PMC228385

[ref39] Vu T. T. T. Hoang T. T. H. Fleischmann S. Pham H. N. Lai T. L. H. Cam T. T. H.. (2022). Quantification and antimicrobial resistance of *Vibrio parahaemolyticus* in retail seafood in Hanoi, Vietnam. J. Food Prot. 85, 786–791. doi: 10.4315/JFP-21-444, PMID: 35226753

[ref40] Wang R. Zhong Y. Gu X. Yuan J. Saeed A. F. Wang S. (2015). The pathogenesis, detection, and prevention of *Vibrio parahaemolyticus*. Front. Microbiol. 6:144. doi: 10.3389/fmicb.2015.00144, PMID: 25798132 PMC4350439

[ref41] Wu X. F. Xu D. S. Ji L. (2021). Surveillance results of foodborne diseases in Huzhou, Zhejiang, 2018-2020. Disease Surveillance. 36, 958–962. doi: 10.3784/jbjc.202105150270

[ref42] Xie T. Xu X. Wu Q. Zhang J. Cheng J. (2016). Prevalence, molecular characterization, and antibiotic susceptibility of *Vibrio parahaemolyticus*from ready-toeat foods in China. Front. Microbiol. 7:549. doi: 10.3389/fmicb.2016.00549 27148231 PMC4839030

[ref43] Yan W. Ji L. Xu D. Chen L. Wu X. (2020). Molecular characterization of clinical and environmental *Vibrio parahaemolyticus* isolates in Huzhou, China. PLoS One 15:e0240143. doi: 10.1371/journal.pone.0240143 33007026 PMC7531842

[ref44] Yan W. Shen Y. H. Xu D. S. (2021). Etiologic characteristics of *Vibrio parahaemolyticus* stains in Huzhou of Zhejiang in 2019. Chinese J Food Hygiene. 33, 74–78. doi: 10.13590/j.cjfh.2021.01.015

[ref45] Yuxian L. Xiufeng L. Fanbing C. Zhiwei C. Haimei Y. Jieru P.. (2023). Genomic characteristics analysis on *Vibrio parahaemolyticus* isolated from food-borne diseases outbreaks in Fuzhou, Fujian province. Disease Surveillance 38, 548–553. doi: 10.3784/jbjc.202207120318

[ref46] Zhang P. Wu X. Yuan R. Yan W. Xu D. Ji L.. (2022). Emergence and predominance of a new serotype of *Vibrio parahaemolyticus* in Huzhou, China. Int. J. Infect. Dis. 122, 93–98. doi: 10.1016/j.ijid.2022.05.023, PMID: 35568367

[ref47] Zhao L. Chen H. Didelot X. Li Z. Li Y. Chen M.. (2019). Co-existence of multiple distinct lineages in *Vibrio parahaemolyticus* serotype O4:K12. Microb Genom. 6:mgen000287. doi: 10.1099/mgen.0.000287, PMID: 31584869 PMC8116679

